# The Ontological Praxis Between Disaster Studies and Demography—Extension of the Scope

**DOI:** 10.1007/978-3-030-49920-4_13

**Published:** 2020-09-18

**Authors:** Dávid Karácsonyi, Andrew Taylor

**Affiliations:** 1 Geographical Institute, Research Centre for Astronomy and Earth Sciences, Hungarian Academy of Sciences, Budapest, Hungary; 2 Northern Institute, Charles Darwin University, Darwin, NT Australia; 3 Faculty of Life and Environmental Sciences, Askja, University of Iceland, Reykjavik, Iceland; 4 Geographical Institute, CSFK Hungarian Academy of Sciences, Budaörsi út 45, Budapest, 1112 Hungary; 5 Northern Institute, Charles Darwin University, Darwin, Ellengowan Dr, Casuarina NT 0810 Australia

**Keywords:** Disaster-demography nexus, Climate change, Urban vulnerability, Geographic possibilism, Mass displacement

## Abstract

This chapter serves as a summary of the learnings from the present volume and an extension of the scope on disaster-demography nexus. We outline the benefits of exploring the disaster-demography nexus and develop a categorisation summarising seven different approaches to the interlink of disasters with demography from examinations of existing literature. These are: disaster impacts on population, measuring vulnerability, mass displacement, spatial-regional approach, climate change, urbanisation and an applied approach. These seven approaches are our attempt to highlight the complex and multifarious nexus between demography and disasters which may not simply be linked to vulnerability. It is recognised that others may separate or merge some of these approaches in different ways.

## Introduction

This chapter serves as a summary of the learnings from the cases presented in this volume and an extension of the scope on disaster-demography nexus. We overviewed in Chap. 10.1007/978-3-030-49920-4_1 the two perspectives on a disaster; the vulnerability school (social embeddedness) and the holistic school (non-routineness). While we stressed in Chap. 10.1007/978-3-030-49920-4_1 that the disaster-demography nexus should immanently be part of the ‘social embeddedness’ perspective, the majority of existing demography studies has a’non-routineness’ outlook on disaster, and feature the disaster-demography nexus through population change as a consequence to disaster. Even the term ‘demography of disasters’ reflected demographic outcomes when it (according to our knowledge) first appeared as the title of the work by Smith (1996); *Demography of Disasters: Population estimates after Hurricane Andrew*.

The nexus was also observed by Schultz and Elliott (2012) in a literature summary of studies of local demographic consequences of disasters in the USA dating back to the early 1980s. In addition, the edited volume by Kurosu et al. (2010) provided a wide range of case studies on demographic responses to environmental crises in the past, such as famines and weather fluctuations in rural societies and epidemic diseases (including smallpox and Spanish flu). However, this volume failed to summarise findings from the cases and provide their theoretical implications for the disaster-demography nexus. There are a plethora of case studies on the disaster-demography nexus in developing countries, especially those concerned with climate change-induced mass relocations. The summarising works, however, are mostly policy-oriented documents with a few exceptions (see for instance Martine and Schensul 2013). This gap in the literature on summarising the multifarious approaches on the demography-disaster nexus was partially filled by two theoretical papers by Donner and Rodríguez (2008) and Hugo (2011) which focused on vulnerability, migration and climate change.

On that basis, the present book and this chapter are not the first attempt to provide a framework on the disaster-demography nexus. Still, our ambition is to traverse the disaster-demography nexus from both ‘non-routineness’ (holistic) and ‘social embeddedness’ (vulnerability) perspectives. These two perspectives cover various forms of the disaster-demography nexus, and, based on the existing literature, we could discern seven different subthemes which are summarised in the following paragraphs and in Table 13.1.

**Table 13.1:** Demographical approaches of disasters

Approaches	Keywords	References	Chapters
Disaster impacts on population incl. disaster epidemiology	Population enumeration, death toll, health impacts fertility/migration response community, regional, country impacts delayed or indirect impacts of disaster: disaster epidemiology increased suicide rate caused by post-traumatic stress	Frankenberg et al. (2014) Nobles et al. (2015) Oliver-Smith (2013) Lindell (2013) Bourque et al. (2007) Veenema et al. (2017) Lechat (1979)) Noji (1995) Briere and Elliott (2000) Krug et al. (1999)	2, 3, 5, 7
Measuring vulnerability (demography is a root cause)	Age, gender, ethnic and social composition of the population, disaster affected special groups—such as disabled people, females, children, elderly people or people on move, refugees, tourists people living under hazard risk adaptive capacity	Malone (2009) Flangan et al. (2011) Fothergill et al. (1999), Fothergill and Peek (2004) Wisner et al. (2004) Bolin (2007) Enarson et al. (2007) Orum et al. (2014) Donner and Rodríguez (2008) Friedsam (1960) Jia et al. (2010) Stough and Mayhorn (2013) Peacock et al. (1997) Zhou et al. (2014))	4, 8, 6
Mass displacement	Forced migration, displaced communities, social cohesion and (local) identity	Oliver-Smith (2013) Naik et al. (2007) Cernea and Guggenheim (1993) Cernea (2004) Gray and Mueller (2012) Levine et al. (2007)	2, 11
Spatial/ regional approach	Development inequalities, scales between community and global	Schultz and Elliott (2012) World Bank (2005) Naik et al. (2007)	2, 4, 11
Climate change	Climate change-induced migration, vulnerability	Oliver-Smith (1996, 2012, 2013) Lavell et al. (2012) Lavell and Ginnetti (2013) de Sherbinin et al. (2011) Bouwer (2011) Martine and Schensul (2013)	8
Urbanisation	Urban concentration and their risks and vulnerabilities to hazard events	Gencer (2013) Armenakis and Nirupama (2013)	6, 9, 10, 12
Practical-applied	Demographic techniques in emergency assessment substitutional practices	Kapuchu and Özerdem (2013) Robinson et al. (2003) Wilson et al. (2016) Brown et al. (2001)	2, 3

## Seven Approaches of Disaster-Demography Nexus

The first and the most commonly used approach to the disaster-demography nexus focuses exclusively on the *consequences from disasters*. *The demography of disasters* by Frankenberg et al. (2014) defined how demographers should study disasters and the essay gives a summary on the various ways disasters affect demographic processes. For instance, a simple demographic technique of measuring disaster impacts is the enumeration of the casualties. Lindell (2013, p. 4) provided the demographic balance equation for this enumeration, basically using the population number before and after the disaster event, subtracting natural population increase and migration to obtain the difference to approximate the impact from the disaster. More complex methods are elaborated by Nobles et al. (2015) to investigate the fertility and natural reproduction response, in particular for baby booms and replacement fertility after disasters. In Bourque’s et al. (2007), understanding a disaster is an unanticipated mortality shock; hence, the subject of demographic analysis is the response to mortality within the community where births represent renewal and the return to ‘normal’. It is important to add that, according to Naik et al. (2007), studies such as these mostly focus on developing countries because usually there are higher death tolls during disasters compared to developed ones.

Disaster epidemiology can be also understood as part of ‘consequences from disasters’, we discussed in the previous paragraph. It should be added that disaster epidemiology and its demographic consequences have been well discussed in the literature (see Lechat 1979; Noji 1995). Recently, Veenema et al. (2017) provided a systematic review on studies related to the climate change-induced hydrological and meteorological hazard events which caused epidemics through lack of access to drinking water. According to Bissel (1983), epidemic diseases appear several months after hazard events because of crowded and inappropriate temporary housing or water transmitted pathogens. These ‘delayed’ deaths are often excluded from the disaster death toll. In addition to post-disaster epidemics, increased suicide rates are also apparent 3–4 years after disaster events and are related to the post-traumatic stress (Krug et al. 1999), distorted life courses or failed, delayed post-disaster recovery.

By extension, demographic consequences from epidemics can be considered as part of a wider disaster-demography nexus, for example, HIV altered demographic trajectories in southern African countries (Nicoll et al. 1994; Gould 2005). The presence of infectious diseases such as HIV, plague, cholera and Ebola (see the 2014 Ebola emergency in Western Africa, Briand et al. 2014) can be explained by poor governance, education, low living standards and lack of modern medicine in the developing world and hence by global social and spatial inequalities. Still antibiotic resistance (WHO 2018), and changing social attitudes (for example, anti-vaccinationism) challenge and may impact demography of developed nations in the future (Kata 2010; Casey 2015).

### Text Box 13.1 the Relationship of Violent Conflicts to the Disaster-Demography Nexus

Some may think about a third angle of the ‘consequences from disaster’ approach as well, which, instead of being part of the holistic school, is related to the social vulnerability paradigm. This third angle may be related to the demographic consequences of malfunctions in society, particularly to economic crises, violence and ‘bad governance’ (Moore 2001). In Wisner’s and his colleague’s (2004) radical embeddedness perspective, all disasters have a root cause in societal failures related to inequalities and spatial exclusion, and hence, violent conflicts are also considered as disasters. But we argue these crises (ethnic cleansings, genocides, sabotages, terrorist actions and violent crimes) are not immanently part of the disaster-demography nexus. As an illustration for the reason for this position, we take the example of outmigration from urban crime hotspots (Foote 2015) which in the *Wisnerian* logic would be considered as part of disaster-demography nexus since violent crime is a kind of malfunction in society. But studies on crime are indeed far from the disaster study field and likely to be related to social sciences other than disaster studies, particularly to criminology, sociology and urban studies. The notion ‘*famine demography*’ by Dyson and Ó Gráda (2002) does not reflect a direct link to disaster. Study of famines has a much stronger link to other fields such as history and economics.

It should be added however that there are often interactions between a violent societal downturn and a coincident natural hazard event which increase population impact which reflects the complexness of a disaster (Robinson 2003; Barton 2005; Cutter 2005). For instance, the *GortaMór* (The Great Irish Famine) killed around one million people and caused mass emigration of the Irish population from Ireland to North America during 1845–1849 (Dyson and Ó Gráda 2002) where the British laissez-faire capitalism, the dependence on one food source and the land rent system interacted with the potato blight. Disasters other than famine are also likely to occur because of violence and war, such as the 1918–1920 Spanish flu pandemic following World War One which caused more death than the war itself (Johnson and Mueller 2002). The 1953 North Sea flood in the Netherlands caused more than 2 000 deaths and was a result of dilapidated state of physical flood defence and warning systems due to the consequences of World War Two and coinciding with an extreme high spring tide and storm surge (Hall 2013).

In some cases, natural hazard events interacting with violent societal downturns are (and were) used to cover up responsibilities for these events (Smith 2014). This is illustrated by the scientific dispute on the causes of high death tolls among indigenous peoples during the colonisation era in several countries, when ethnic compositions of entire continents have been changed. While Crosby (1976) and McNeil (1976) considered a decisive role of the effects of ‘virgin soil epidemics’ for these high death tolls due to lack of immunity of indigenous people to the infectious diseases introduced by Europeans, their view is now strongly disputed (Jones 2003). Recently, most of authors explaining death tolls through genocide (see Lemkin 2012; Curthoys 2005; Jones 2017) and indigenous population decline through loss of livelihoods during colonisation (Smith 1989).

The *applied demography approach* is also strongly affiliated with the ‘consequences from disaster’ paradigm, but, in contrast to disaster epidemics and societal downturns, we conceptualise it as a separate theme (the second one among the seven approaches of disaster-demography nexus summarised in Table 13.1). The applied approach was emphasised by Robinson et al. (2003) who summarised the demographic means in relation to disaster mitigation in their work *Principles and uses of demography in emergency assessment*. The applied demographic techniques in disaster situations can help to measure a disaster’s impact on the affected population. Moreover, according to Lindell (2013), applied demographic methods can be used in every stage of the disaster cycle. Despite the widespread use of demographic techniques to study disasters, Frankenberg et al. (2014) have emphasised that, due to a lack of adequate, spatially and timely detailed data, there are not many studies which would interrelate demography and disasters through mortality and fertility changes to provide an integrated analysis. Hence, Robinson et al. (2003) provided an overview of substitutional procedures to be used for disaster assessment where there is not an adequate dataset. To fill the immediate knowledge gap about the size of affected populations, the area sampling method is usually applied to estimate the numbers impacted in developing countries (Brown et al. 2001). In developed nations, mobile phone location data can also substitute for adequate datasets for disaster impact assessment for population, as it is discussed in Chap. 10.1007/978-3-030-49920-4_2 and by Wilson et al. (2016). Among administrative data, school enrolments can provide an estimate on population displacement (Plyer et al. 2010) as it also presented in Chap. 10.1007/978-3-030-49920-4_3. Longitudinal practices and the application of supplementary data on disaster impacts, for example, housing damages, are also introduced in Chap. 10.1007/978-3-030-49920-4_12.

While the assessment of the demographical consequences of disasters in the previous paragraph has strong links to the non-routineness (holistic) school, the third approach which addresses *demography as root cause* of the disaster is clearly part of the ‘social embeddedness’ perspective. As an illustration for social, or more precisely ‘demographic embeddedness’, Malone (2009) called attention to the importance of using demographic analysis to measure vulnerability. In fact, socially created vulnerabilities are difficult to quantify (James 2012) because they are a combination of different factors (Wisner et al. 2004). Furthermore, Malone (2009, p. 13) provided a method and a group of indicators, including demographic data to measure vulnerability and resilience in the form of a vulnerability-resilience indicators model. Malone suggested to use detailed socio-demographic analysis, for example, population distribution and density, births and mortality in different areas characterised by different livelihoods. These demographic analyses throw light on the social context that allows analysts to see how households are constituted, the elements that affect their functioning and disrupts them. Other similar indexes have been developed recently to measure social vulnerability (see Flanaganet al. 2011; James 2012). As an example, by using a combination of multivariable and spatial analysis in vulnerability assessment, Zhou et al. (2014) used factor analysis to create a complex disaster vulnerability index at a county level for China (2361 units in total) based on population census data. Zhou and his co-authors analysed also the spatial variation of index values obtained from factor analysis by using local and global autocorrelations which helped them to identify vulnerability hotspots to hazard events.

Vulnerability assessment is often part of planning for large industrial investments. As an illustration, Orum et al. (2014) provided an estimate of vulnerability using demographic data of people living in the zones around more than three thousand chemical plants in the USA. They focused primary on social status and ethnicity and found that residents of chemical facility vulnerability zones belong mostly to minority groups (African Americans or Latinos). These populations have higher rates of poverty and cheaper housing, lower incomes, and education levels than the national average. The case of Hurricane Katrina (2005, New Orleans, USA) also drew attention to race and ethnic inequalities in the USA (Bolin 2007 p. 113, or Chap. 10.1007/978-3-030-49920-4_6), because the lower laying flood-prone areas were mostly inhabited by poorer African Americans. According to Peacock et al. (1997), ethnic segregation also occurred during the post-Hurricane Andrew relocation (1992, Florida) which caused social change and put African American communities into a more vulnerable position.

As Peacock’s study suggests, demographic vulnerabilities are not only root causes, they can deteriorate disaster consequences as well. For instance, along with ethnicity (Fothergill et al. 1999; Fothergill and Peek 2004), gender (see Enarson 2000; Enarson et al. 2007, or Chap. 10.1007/978-3-030-49920-4_9) and age composition can make communities more vulnerable. In particular, population ageing in developed countries is establishing age-related vulnerable population enclaves (Fernandez et al. 2002). As an illustration, Isoda (2011) pointed out that, during the 2011 Great East Japan Earthquake and Tsunami, the death toll was higher among the elderly who typically stayed at home when the tsunami hit and were not able to escape. This is because the tsunami hit Japan during working hours, and in contrast to schools and job places, private homes were neither designed to resist the tsunami nor provided with shelters. Consequently, the death toll was in general higher in those rural communities on the coastal areas where the proportion of elderly people in the population were higher. A high death toll among the elderly was also observed in the case of 2008 Sichuan earthquake (Jia et al. 2010). Friedsam (1960) in his early literature review on disasters in the USA and on impacts of World War Two bombings in Europe distinguished direct (older people are more likely to be hit since they have limited mobility) and indirect or secondary effects (lack or poor level of medical treatment for people in need during the emergency). Furthermore, indirect effects include a shortening of life expectancy for people living with diabetes (Fonseca et al. 2009) or for those requiring any kind of regular medical treatments such as haemodialysis. Ironically, despite the higher death tolls among elderly, they are the most likely to be ‘post-disaster returners’ to an area because of their stronger attachment to place through their longer life experience (see Chaps. 10.1007/978-3-030-49920-4_2 and 10.1007/978-3-030-49920-4_10).

Along with static demographic aspects such as age, race and sex composition, we summarised in the previous two paragraphs, dynamic demographic aspects, in particular migration, should be also considered when discussing vulnerabilities. For instance, people on move, such as refugees, internally displaced or tourists are particularly vulnerable to hazards (Robinson 2003; Donner and Rodríguez 2008). Large numbers of tourists were impacted during the 2004 Indian Ocean tsunami, since they had a high concentration in coastal areas during the Christmas high season (Becken et al. 2014). Of course, refugees are generally more vulnerable when compared to tourists because of their social status. According to Naik et al. (2007) excess populations, such as visitors can easily become the ‘forgotten group’ in the course of a disaster because of a lack of response planning (ibid. p. 57).

The vulnerability of people on the move links us to the fourth meta-approach to the disaster-demography nexus which focuses on *migration and mass displacement*. According to Hugo (2008), migration has always been one of the most important survival strategies adopted by people facing disasters. As an illustration to Hugo’s point, King and Gunter in Chap. 10.1007/978-3-030-49920-4_6 highlighted population loss due to post-disaster outmigration in case of New Orleans (Hurricane Katrina), Christchurch (2011 earthquake) and Innisfail (2006 and 2011 cyclones). Lavell and Ginnetti (2013) suggest, the demographic profile of entire regions can be altered over a long period of time as consequence of disaster-induced mass displacements. Furthermore, these mass displacements can be short or long distance, temporary or permanent (Cernea and Guggenheim 1993; Cernea 2004). Regarding research on long-term and long-distance displacements after disasters, Levine et al. (2007) pointed out a gap in the literature due to lack of data and the difficulty to follow up such migration.

Furthermore, the non-spontaneous character of mass displacement is stressed by Oliver-Smith (1996); the relocation or resettlement of disaster-stricken populations is a common strategy pursued by planners in post-disaster reconstruction efforts. Displacement is also selective based on vulnerabilities and the connection between migration and hazard impact is not always clear. As an illustration, Gray and Mueller (2012) brought to the fore that those families impacted directly by a disaster are less likely to move out compared to those in the disaster-prone area not impacted directly. This is because the latter group has the means to fund their move, while the former, who may have lost everything, are ‘stuck’ in the disaster-prone area. It is important to add that displacement is not only related to disaster, it is a broader and more common phenomenon. That is, displacement could take place because of occurrence of a disaster, climate change inducted environmental change (see Chap. 10.1007/978-3-030-49920-4_8), violent conflict or a development project (Oliver-Smith 2013). Furthermore, Oliver-Smith (ibid.) as well as Scudder and Colson (1982) highlighted that development projects cause much more displacements than all disasters combined.

Disaster-induced spatial movement brings the fifth approach to the fore which is the disaster-demography nexus in the *spatial-geographical context*. The spatial-geographical approach has roots in disaster studies in the first half of twentieth century (see White 1945) through the theory of environmental adaptation (Alexander 2001). Environmental adaptation reflects the conviction of *geographic possibilism,* a dominant paradigm of human geography in the first half of twentieth century and itself rooted in the French regional geography (see Vidal de la Blache 1911). The concept of geographic possibilism means that the diversity of the natural environment provides different opportunities and constraints where people react to their environment and make their own choices. These choices, their ways of life (*genres de vie*), are the manifestation of their culture in the Vidalian regional geography.

Geography and space also play an important role in the social embeddedness perspective on disasters as well. But instead of the now outdated geographic possibilism, it is connected to the neo-Marxist critical geography paradigm (see Harvey 1996). Within the embeddness viewpoint, space is understood to be an uneavenly distributed resource because of uneaven population distributions, and hence, the uneaven allocation of resources within societies. Wisner et al. (2004, p. 5) described this as follows: ‘People live in adverse economic situations that oblige them to inhabit regions and places that can be affected by natural hazards’. Wisner et al. (2004) stressed that different groups take risks for advantages voluntarily or involuntarily because of their economic needs. For example, they take risk of landslide to have a house on a slope for a better panorama or they live in a poorly built informal settlement on a slope in an urban area to access better job opportunities. The former highlights increases risks from voluntary actions, while the latter demonstrates the ‘forced’ acceptance of risk for (economic) survival.

Furthermore, according to Cutter (2005, p. 42), the global extent of risks is not equally distributed among all places or among all social groups. This spatial inequality approach is highlighted by Carson and his colleagues in Chap. 10.1007/978-3-030-49920-4_5 that the Great Deprivation (the Swedish famine of 1867–1868) ‘…is seen as the last of the European famines to result from natural events’. However, Carson and his colleagues also highlight that Northern Sweden was a territory for Swedish northern settlement advances encouraged by the vast mineral and forest resources there at that time. Crop failure triggered by the cold summer of 1867 hit especially those northern advance settlements and caused famine there. Wisner’s interpretation would suggest the famine to be a result of northern advance into sparsely populated areas rather than the cold summer (the natural element in the disaster). But Carson also stressed that improving food supply chains and reducing reliance on local food production helped northern sparsely populated territories to avoid famines later on; hence, the famine was the result of unfavourable economic patterns at the time.

According to Lavell and Ginnetti (2013), economic development, better technologies and improving living standards are reducing vulnerability on a global basis. Alexander (2005, p. 32) stressed, however, there is an endless resurgence of vulnerability, because of growing socio-economic inequalities and polarisation throughout the world. Alexander’s perspective was also echoed by Naik et al. (2007). Accordingly, disasters have a disproportionate effect on developing countries because of poor quality of construction and less compliance with building codes, and absence or non-application of land registration and other regulatory mechanisms (ibid. p. 19). Furthermore, according to Naik et al. (2007), there is a significant difference in the impact of disasters on developing and developed countries in terms of the type of loss: data show a higher death toll in developing countries compared to developed countries, but absolute economic losses are greater in developed countries because of higher concentration of economic assets in the area. This was also supported by Robinson (2003 p. 5), who stated, that between 1991 and 2000, 3 million people were killed by disasters, while only 2% of them were from highly developed countries while 60% were from Africa.

The examples on spatial-geographical context we highlighted in the previous paragraphs showed that space allocation has a slightly different meaning depending on its ‘local’ or ‘global’ connotation. But Cutter and Wisner have tended to use these interchangeably in their arguments when explaining the role of space allocation in vulnerabilities. But the scale is important, because the former brings to the fore local social inequalities, the latter features global geographical diversity and inequalities among nations. Hence, the size of areal unit under consideration can influence the phenomenon we are observing (see Chap. 10.1007/978-3-030-49920-4_5 and Koch and Carson 2012 on the modifiable areal unit problem).

On a global scale, spatial variety of the natural environment provides different types of environmental opportunities and risks; hence, global assessment on natural hazard hotspots has a high priority for development and aid agencies (Nadim et al. 2006; Strömberg 2007). For example, the World Bank (2005) conducted a study on natural hazard hotspots to estimate GDP losses and mortality as a consequence of disasters in various countries. The study distinguished and analysed eight types of disasters based on their ‘natural’ characteristics; drought, storm, flood, earthquake, volcano, heat wave, landslide and wildfire. Accordingly, the World Bank used the terms single hazard hotspots and multiple hazard hotspots and investigated the exposure to these risks and vulnerabilities for different countries. Another example is the work by Schultz and Elliott (2012) which used census and hazards database to estimate the demographic consequences of disasters at a county level in the USA. They found a positive correlation between cumulative hazard impact during the 1990s and changes in local population numbers. Further examples of hazard risk assessments are the estimates of global flood risk (Winsemius et al. 2013), global landslide and avalanche risk (Nadim et al. 2006), tropical cyclone risk (Peduzzi et al. 2012) and their effects on population and GDP.

Despite the demand for natural hazard hotspot assessments, there are a relatively small number of such studies and they are absent in sociology based disaster studies. This is likely because the ‘social embeddedness’ disaster school denies the concept of *geographic possibilism* discussed earlier. Despite this, the spatial-environmental diversity is important especially in the course of emerging climate change (or more explicitly the, climate emergency faced globally) which highlights the low resilience of our political, social and technical systems, constraints and limitations of human society when coping with ‘nature’.

Indeed, the *impact of and adaptation to climate change* requires special attention, which forms the sixth approach of demography-disaster nexus. Climate change connects disasters, spatial-geographical diversity and migration. As an illustration, according to de Sherbinin and his co-authors (2011), climate change-induced mass relocation is a politically disputed and socially sensitive adaptation strategy but seems to be unavoidable in the very near future. Adding to this, Oliver-Smith (2013) summarised the potential forces which could lead to mass displacement during climate change impacts; such as evacuation because of rapid onset events (typhoons, floods), slow onset drivers for forced migration (drought, desertification), or displacement from climate change mitigation projects (resettlement from coastal areas, large constructions as reservoirs or coastal defence dams).

While climate change may have extensive impacts through forced migration, the international dimensions of this relationship have been neglected until recently (Hansen et al. 2012; Jankó et al. 2018). Hugo (2008) suggests this is because such events have affected mostly developing nations. Adding to this, Bouwer’s (2011) literature review spotlighted that losses caused by climate change are not significant so far but will be significant in the near future. Extending this, Zander and her colleagues suggest climate change adaptation-induced migration (intention to migrate because of climate change related heatwaves) is also present within developed nations, such as Australia (see Chap. 10.1007/978-3-030-49920-4_7). However, others (McLeman and Hunter 2010; Carson et al. 2016) have stressed that weather-induced mass migration in developed countries is mostly seasonal-temporal. These include the *snowbirds* in North America and the *grey nomads* in Australia, who seasonally move between the tropical–subtropical and temperate zones of their respective continents.

Lavell and Ginnetti (2013) and Hugo (2011) draw attention to the fact that to date most climate change migration caused by environmental change has occurred within national boundaries. According to McLeman and Hunter (2010), these climate change-induced internal and intra-regional moves (within a region of one country) were up to now temporary. Hugo (2011) stressed that the occurrence of climate change-induced extreme weather events is just one factor among several others influencing migration decisions. Hence, a natural hazard alone does not lead automatically to displacement (Piguet et al. 2011 p. 23).

Furthermore, according to IOM (2012), climate change-induced migration can be both a challenge and a solution for the problems, as people move to less affected areas. According to Piguet et al. (2011), migration is an adaptation strategy which should not be considered as a negative outcome to be avoided. For example, according to Naik et al. (2007), environmental migration can affect development not only negatively (through the exodus of highly skilled people, loss of workforce, brain drain and so on), but emigration can ease pressure on the environment, while remittances and returning experienced people can also boost the economy and promote development goals. Additionally, Naik et al. (2007) focused on how migratory flows and migrant communities are impacted by disasters, and how kinship and support from diaspora affected migrant communities in the aftermath of disasters (through, for example, aid and technical assistance).

As an illustration of the potential scale of climate change-induced migration, Lavell and Ginnetti (2013) estimated the likelihood of disaster-induced displacement and quantified the number of people at displacement risk using a probabilistic risk model. Their estimate showed that almost 3 thousand per million people are displaced annually in Central America and the Caribbean as a result of climate change, equating to 300 thousand per year. In fact, the potential scale of future environmental-induced migration is the subject of debate and its impacts will be very different around the world (Piguet et al. 2011) because impacts are determined not just by the absolute exposure and the size of exposed population but by their conditions of resilience and vulnerability as well (Oliver-Smith 2012). Based on these complex interactions, Oliver-Smith (2012) questioned whether can we really speak about migration directly induced by climate change? Probably the direct connection is present in the case of small nations of the Pacific suffering from direct effects of sea level rise and considered as first victims to climate change (Farbotko and Lazrus 2012). But according to Hugo (2011), demographic hotspots (places, countries with population booms) and climate change hotspots overlap in space (these hotspots are in Africa, in South and Southeast Asia and in Central America and the Caribbean), which is generating a complex interactions with migration and will cause increased mobility in future affecting developed nations as destinations as well (Reuveny 2007).

The interaction of climate change, population booms and spatial inequalities has fuelled rural to urban migration and the urbanisation boom in developing countries (Hugo 2011). Hence, according to Gencer (2013), disaster studies should pay special attention to urban vulnerability and disaster risk reduction, linking disasters, the global trend of urbanisation (Clark 1996; Seto et al. 2011) and climate change. So, the seventh approach to the disaster-demography nexus is related to *urban vulnerability*. Donner and Rodríguez (2008) stressed that increasing urban vulnerabilities are particularly evident in rapidly growing coastal megacities of developing countries such as Jakarta, Dhaka and Lagos (see Tacoli et al. 2015; Di Roucco et al. 2015). More generally, rural to urban migration means that people arriving to high hazard risk urban areas from rural areas are generally characterised by low economic opportunities but also a lower probability of hazard impacts (Hugo 2011). For example, empirical evidence suggests per capita death tolls are higher from earthquakes in urban areas compared to rural regions (Donner and Rodríguez 2008). Wisner and colleagues (2004) suggest urbanisation as major factor in the growth of vulnerability, particularly for low-income families living within squatter settlements in developing countries. These informal settlements are exposed to physical vulnerabilities due to their construction practices or location in hazard risk areas. In these informal settlements, social vulnerability and exclusion are strongly related to hazard risk exposures such as floods (Amoako et al. 2018) or landslides (Chardon 1999; Alves and Ojima 2013). As a result, urbanisation and rapid population growth together have led to the concentration of population in hazard prone urban areas and hence put more people at risk.

While it seems obvious that the urban vulnerability context is related primarily to developing countries, that is not exclusively the case. For example, pandemics can spread rapidly across global cities of developed nations as well (Alirol et al. 2010; Grais et al. 2003) such as during the 2002–2003 SARS coronavirus (in East Asia) and during the 2009 H1N1 flu virus (in North America) epidemics (McLafferty 2010). Armenakis and Nirupama (2013) highlighted that risks of technological disasters related to certain industries (nuclear, chemical or biotech facilities, gas supply systems) are high in urban zones of developed countries as well. To cope with these hazard risks, properly designed rapid evacuation systems are needed based on geography, population sizes, distributions, compositions and vulnerabilities (Kendra et al. 2008). Adding to this, Singh in Chap. 10.1007/978-3-030-49920-4_10 has emphasised there are more complex and interlinked (and hence vulnerable) lifeline networks under risks with growing urbanisation (see also Tielidze et al. 2019). Furthermore Murao in Chap. 10.1007/978-3-030-49920-4_12 pointed out that cities can be considered as engineering ‘products’; however, unlike other products, they have never been tested before people start using them during their everyday life. In Chap. 10.1007/978-3-030-49920-4_9, Barnes argued that urban landscape, engineering, social and community aspects are linked together in urban disaster resilience and that has different outcome for females. Additionally, there is a need to develop ‘age-friendly cities’ (Buffel et al. 2012), because of the growing number of elderly in urban areas of developed countries representing a highly vulnerable group to disaster risk as well (Donner and Rodríguez 2008). Based on the various aspects, we featured here on urban vulnerability it is clear that this approach of disaster-demography nexus is strongly linked to other approaches such as demographic vulnerability and climate change as well.

## Conclusions

In this chapter, we have laid-out the links between disasters and demography evident in the field of disaster studies and plotted major historical paradigm changes in the field. Of course, this classification is subjective and others may separate or merge some of these approaches in different ways. The collective case studies in this volume further expand the links by highlighting the complex and multifarious nexus between demography and disasters which may not simply be linked to vulnerability. While, for example, demographic conditions prior to a disaster may be the reason for high impacts (for example, loss of life); it may also reflect longer-term and more localised structural changes in the demography of towns or regions. In terms of demographic consequences for disasters, as some chapters in this book noted (for example, Chaps. 10.1007/978-3-030-49920-4_2 and 10.1007/978-3-030-49920-4_5), disasters may be an agent for speeding up pre-existing demographic trends, such as rural to urban migration. The demographic profile ‘left behind’ may consequently be quite different to pre-disaster but, without detailed examination of pre-disaster demography, it would be easy to suggest that the disaster fundamentally ‘caused’ a new demographic structure at the local level of impacts.

Taking a wider perspective of demography, which goes beyond the statistical analysis of populations, enables us to depart from the classical scope of demographically rooted disaster studies in which the disaster is traditionally singularised as a root cause for demographic shifts (the non-routine event approach). In this volume, we attempted a broader demographic purview in order to extend disaster science research.
